# Treatment of Phthisis by Subcutaneous Administration of Carbolic Acid

**Published:** 1886-02

**Authors:** 


### TREATMENT OF PHTHISIS BY SUBCUTANEOUS ADMINISTRATION OF CARBOLIC ACID.

Dr. Filleau, of Paris, reports that he has derived excellent results by the employment of carbolic acid hypodermically in the treatment of phthisis pulmonalis.

Being a firm believer in the parasitic origin of phthisis, he searched for an antiseptic or germicide which could be injected into the blood without harm to the patient. Iodoform was tried, and failed. But chemically pure carbolic acid seemed to answer all requirements. It is easily mixible with water, can be injected without pain, and never causes abscess or phlegmosis. Moreover, it has been demonstrated by Paul Bert that carbolic acid is eliminated by the lungs as well as by the kidneys, thus reaching the favorite seat of the bacilli, and acting like an antiseptic lotion. It was further observed that the *bacillus tuberculosis* was quickly destroyed by very weak solutions of this antiseptic.

The formula for the preparation of Dr. Filleau's solution is—

| | | |
|---|---|---|
| ℞ | Acid, Carbolic, c. p., - - - | 1 pt. |
| | Glycerini Puri, - - - | 4 pts. |
| | Aq., Destillat, - - - - | 95 pts. |

M. Sig. 100 minims once a day or every other day, according to the case.

The carbolic acid must be perfectly pure. That having a rose color should never be employed. The treatment should be continued persistently unless symptoms of intoxication appear, in which case the medication should be dropped.

Dr. Filleau has employed this treatment with very satisfactory results for two years. Several of his patients have been exhibited in the Hospital Cochin to the profession. He summarizes his theory and treatment as follows :

" 1. The parasitic origin of tuberculosis being admitted, carbolic acid, c. p., must be considered the best antiseptic to be employed in the treatment of tubercular diseases.

"2. Carbolic acid is the only germicide which can be administered subcutaneously for a long time without danger.

"3. The safety and toleration of carbolic acid given hypodermically have been thoroughly demonstrated.

"4. By means of this treatment, the general condition of the patient is rapidly improved, and the local lesions are favorably modified.

"5. The treatment in every case should be persistently carried out."

